# Multi-Modal Analysis of Resting-State fMRI Data in mTBI Patients and Association With Neuropsychological Outcomes

**DOI:** 10.3389/fneur.2021.639760

**Published:** 2021-05-17

**Authors:** Jian Shi, Jing Teng, Xianping Du, Na Li

**Affiliations:** ^1^Department of Spine Surgury, The Third Xiangya Hospital, Central South University, Changsha, China; ^2^School of Control and Computer Engineering, North China Electric Power University, Beijing, China; ^3^Department of Mechanical and Aerospace Engineering, Rutgers University, Piscataway, NJ, United States; ^4^Department of Radiology, The Third Xiangya Hospital, Central South University, Changsha, China

**Keywords:** mild traumatic brain injury, rs-fMRI, neurological disorders, regional homogeneity, clinical and imaging assessment, multi-modal analysis

## Abstract

Various cognitive disorders have been reported for mild traumatic brain injury (mTBI) patients during the acute stage. This acute stage provides an opportunity for clinicians to optimize treatment protocols, which are based on the evaluation of brain structural connectivity. So far, most brain functional magnetic resonance imaging studies are focused on moderate to severe traumatic brain injuries (TBIs). In this study, we prospectively collected resting state data on 50 mTBI within 3 days of injury and 50 healthy volunteers and analyzed them using Amplitude of low-frequency fluctuation (ALFF), Regional Homogeneity (ReHo), graph theory methods and behavior measure, to explore the dysfunctional brain regions in acute mTBI. In our study, a total of 50 patients suffering <3 days mTBI and 50 healthy subjects were tested in rs-fMRI, as well as under neuropsychological examinations including the Wechsler Intelligence Scale and Stroop Color and Word Test. The correlation analysis was conducted between graph theoretic parameters and neuropsychological results. For the mTBI group, the ReHo of the inferior temporal gyrus and the cerebellum superior are significantly lower than in the control group, and the ALFF of the left insula, the cerebellum inferior, and the middle occipital gyrus were significantly higher than in the control group, which implies the dysfunctionality usually observed in Parkinson's disease. Executive function disorder was significantly correlated with the global efficiencies of the dorsolateral superior frontal gyrus and the anterior cingulate cortex, which is consistent with the literature: the acute mTBI patients demonstrate abnormality in terms of motor speed, association, information processing speed, attention, and short-term memory function. Correlation analysis between the neuropsychological outcomes and the network efficiency for the mTBI group indicates that executive dysfunction might be caused by local brain changes. Our data support the idea that the cerebral internal network has compensatory reactions in response to sudden pathological and neurophysiological changes. In the future, multimode rs-fMRI analysis could be a valuable tool for evaluating dysfunctional brain regions after mTBI.

## Introduction

Mild traumatic brain injury (mTBI) makes up about 75% of TBI cases (1). Furthermore, the actual incidence of mTBI is estimated to be even higher than the mTBI cases reported by hospitals (2). Hence, much effort has been invested in understanding mTBI, which is usually difficult to diagnose but could lead to serious sequelae, in order to improve brain health. One limitation in understanding and treating mTBI is that many mTBI patients do not receive special interventions in hospitals (3), partially due to the fact that mTBI patients do not show obvious abnormalities through conventional CT and MRI imaging. Meanwhile, many patients with mTBI develop both acute and chronic neurological symptoms and cognitive defects (4, 5). Further, retrospective studies have shown that the incidence of neuropsychiatric disorders in patients with previous mTBI in their younger age is higher compared with those without previous mTBI (6). To better help patients during the acute phase, which provides a window for interventions that may treat mTBI, more studies on the abnormality of brain network and the neurocognitive function in the acute phase are needed.

Resting-state functional magnetic resonance imaging (rs-fMRI) reflects time synchronization of several brain regions when the brain is in the resting state without a specific task. It can also show a significant amount of spontaneous neuronal activity, and it may reveal cognitive impairment caused by mTBI (7). The main advantage of rs-fMRI is its capability of finding abnormal functional connections, even when structural deficits are absent. And new methods based on graph theory could be used to compare the patients with brain injury in the acute phase and even half a year later (8). There have been extensive reports of changes in connectivity in patients with moderate to severe TBIs, Alzheimer's disease, dementia, anorexia nervosa, and schizophrenia (9–13), which is of great significance.

In this study, rs-fMRI data and T1 anatomical image data from mTBI patients and a control group were collected. The global and local graphic parameter differences of functional network connection between the mTBI group and the control group were compared by rs-fMRI (14). First, the ReHo and ALFF between the two groups were compared, and then the changes of local and global efficiency for mTBI patients at the acute phase were calculated by analyzing rs-fMRI data using the graph theory method. The standard neuropsychological method was used to evaluate the changes of executive function for mTBI patients in the acute phase, and the correlations between executive network damage and mTBI patients' neuropsychological assessment were explored.

## Methods and Materials

### Experimental Subjects

Head trauma patients treated in the emergency department of the third Xiangya Hospital of Central South University from April 2014 to March 2020 were recruited. Healthy volunteers with matching age, gender, and education level were also recruited.

All the subjects eligible to participate in this study meet the following inclusion and exclusion criteria. The study was approved by the IRB of Third Xiangya Hospital, Central South University, and the study on humans was carried out in accordance with the relevant Measures for the Ethical Review of Biomedical Research Involving Humans (China, 2016). All participants in the study signed an informed consent form. Group characteristics are shown in Table 1.

**Table 1:** Group characteristics.

	**mTBI group (*N* = 50)**	**Control group (*N* = 50)**	***P*-value**
Sex (M/F)	25/25	25/25	N/A
Age (yr)	24.3 ± 5.2	24.1 ± 4.9	0.58
Height (cm)	169.5 ± 10.1	170.1 ± 10.0	0.41
Weight (kg)	70.4 ± 10.9	69.9 ± 9.2	0.94
Mechanism of Injury	Traffic accident (28) Activity of daily Living (9) Sport Activity (13)	N/A	N/A
GCS score	14 (26) 15 (24)	N/A	N/A
LOC	<30 min	N/A	N/A

The inclusion criteria (all required) specified that patients: (1) The GCS score is between 13 and 15 points; (2) Loss of consciousness (LOC) lasted <30 min; the subject had post-traumatic amnesia (PTA) of <20 h or had an alternation in the mental state (e.g., disorientation, bewilderment or confusion); (3) No intracranial hematoma showed up on a CT scan; (4) Injury lasted <3 days; (5) The patient did not have any other serious physical injuries (such as multiple fractures, etc.); (6) The patient is right-handed and aged from 18 to 55 years old. The exclusion criteria included (1) Having a history of head trauma previously; (2) Intracranial hematoma or cerebral contusion that showed on CT; (3) Having neurological, psychiatric, or psychological illness; (4) Abuse of drugs, alcohol, or tobacco; (5) Being a non-cooperator because of mentally disturbed; (6) Having had claustrophobia; (7) Having had metal implants in the body; and (8) Pregnancy or plans to become pregnant.

**Figure 1:**
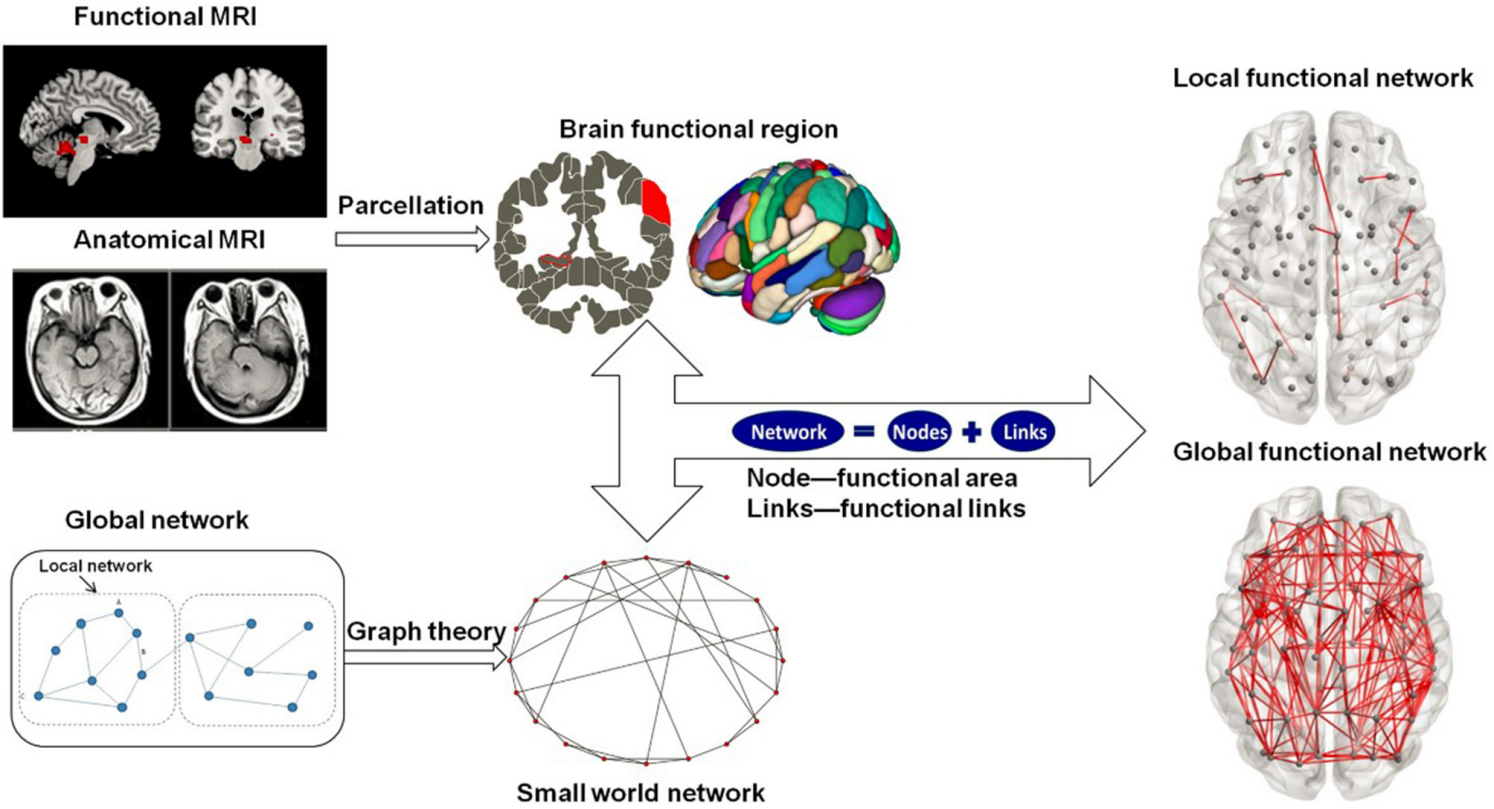
Overview of image analysis steps.

For the selected patients and control group, the same analysis procedure (Figure 1) was adopted to analyze local and global functional network parameters. All the procedures have been approved by the university ethical committee on human subject research.

### Neuropsychological Assessment, PSQI, and Treadmill Test

In the acute phase (3 days after injury), patients received a set of neuropsychological assessments in clear consciousness and emotionally stable condition. The Wechsler Adult Intelligence Scale (WAIS), the most widely used tool for intelligence and neuropsychological assessment for adults aged 16 or older (15), was chosen for this study, in which the Stroop color-word test (S-CWT) is used in the evaluation of craniocerebral trauma (16, 17), was also adopted. Furthermore, the Pittsburgh Sleep Quality Index (PSQI) and the treadmill test results were collected to analyze the cognitive dysfunction relevance between the clinical neuropsychology and functional MRI; the detailed data are shown in Table 2.

**Table 2:** The neuropsychological assessment, PSQI, and treadmill test results of the mTBI group and the control group.

**Behavior and Information**	**mTBI**	**Control group**	***P*-value**
Knowledge	11.91 ± 1.95	12.33 ± 2.30	0.53
Number span	13.00 ± 2.81	12.722 ± 3.25	0.77
Drawing and filling	9.18 ± 2.01	9.944 ± 2.555	0.30
IQ	105.91 ± 12.32	107.222 ± 9.32	0.61
Digit sign test	58.773 ± 8.97	68.611 ± 9.70	0.00028^*^
Stroop-Word	22.415 ± 4.36	19.735 ± 4.20	0.08
Stroop-Color	35.652 ± 4.71	31.575 ± 5.72	0.018^*^
Stroop-Interference	61.061 ± 14.68	53.312 ± 10.20	0.18
Sleep (PSQI)	6.355 ± 10.61	3.92 ± 9.19	0.074
Sport (Treadmill test)	4.77 ± 9.24	5.26 ± 8.72	0.048

* *p < 0.05*.

### Image Acquisition

The PHILIPS Ingenia 3.0T MRI of the third Xiangya Hospital in Central South University was adopted for the use of image scanning. It is equipped with a 15-channel head coil. All patients (within 3 days after injury for the mTBI patient group) received a high-resolution 3D sequence, a T2-FLAIR sequence, and an rs-fMRI sequence scan. The detailed sequence parameter was the same as with our previous study (18). During scanning, subjects were placed in a supine position on the bed, on which foam pads constrained the head to limit head movements. During the process, subjects were instructed to close their eyes and stay awake in the resting position without any cognitive tasks.

### Rs-fMRI Pretreatment

The BOLD data were preprocessed using AFNI software and MATLAB 2013a. The T1-weighted structure image was fed into the MNI (Montreal Neurological Institute) template using the uniform standardized spatial segmental method. As head motion affects the quality of rs-fMRI data during the scan, a method was developed to detect and remove the data above the RMS error of the whole brain (19). Then the slice timing, realignment, co-registry, normalization, smoothing, filtering, and segmenting were processed before the section Reho and ALFF.

### ReHo and ALFF

**Figure 2:**
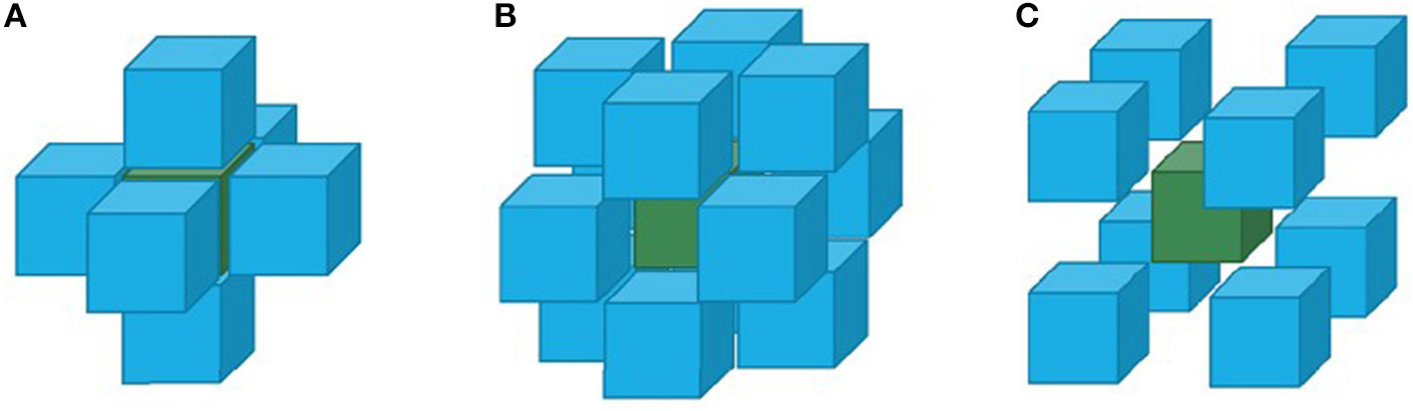
Three different situations of adjacent voxels analyzed by the ReHo method: **(A)** six adjacent voxels connected with six surfaces; **(B)** twelve adjacent voxels connected with twelve sides; **(C)** eight adjacent voxels connected with eight angles.

Regional homogeneity (ReHo) utilized the Kendall's coefficient of concordance to measure the time series varying the consistency of bold, which present the dysfunctional brain region (Figure 2), the equation was the following:

(1) $$\text{w}=\frac{\sum {\left(\text{Ri}\right)}^{2}-{\text{n}\bar{\text{R}}}^{2}}{\left(\frac{1}{2}){\text{K}}^{2}({\text{n}}^{3}-\text{n}\right)}$$

In which *w* represents the voxel Kendall's coefficient of concordance, *w* ∈ (0, 1); when *w* is closer to 1, the consistency is higher. Here, *n* represents time, and *K* is the voxel number. R_*i*_ is the voxel summary of the *i* time node; $\bar{\text{R}}$ is the average of the R_*i*_. In this study, the ReHo value represents the local consistency of the brain area, while the lower ReHo value indicates the axon nervous disorder in this local brain area.

By analyzing the correlation coefficient of the functional brain areas in different frequency domains, (20) found that frequencies below 0.1 Hz were the main source of functional brain connectivity, and then ALFF was proposed by Zou et al. to reflect the activity of all brain voxels in the resting state (21). This method first removes the linear drift from the preprocessed data, and then the low-frequency signals are filtered out through a band-pass filter. The low-frequency signal is processed by a Fourier transform by taking the square root of the transformed power spectrum; the average value of the lower amplitude of each frequency is obtained to present the activity of each brain region.

The clustering coefficient is a measure of the separation function. The clustering coefficient C_*i*_ of node *i* is defined as the ratio of the number of the actual edges between node *i* and its adjacent nodes and the largest possible numbers of edges among those adjacent nodes, as shown in equation (2),

(2) $${\text{C}}_{\text{i}}\text{=}\frac{{\text{2E}}_{\text{i}}}{{\text{k}}_{\text{i}}({\text{k}}_{\text{i}}-\text{1})}$$

Where E_*i*_ denotes the number of edges between node *i* and all other nodes which have connection with it. K_*i*_ represents the number of nodes directly connected to node *i*. The denominator indicates the maximum number of possible edges between node *i* and its connected nodes. The clustering coefficients range from 0 to 1. Usually, C_*i*_ represents the average of all nodes in the whole network, so the clustering coefficient of the whole network can be expressed as C (*n* represents the number of nodes in the network):

(3) $$\text{C}=\frac{1}{\text{n}}\sum_{\text{i}\in \text{G}}{\text{C}}_{\text{i}}=\frac{1}{\text{n}}\sum_{\text{i}\in \text{G}}\left(\frac{2{\text{E}}_{\text{i}}}{{\text{k}}_{\text{i}}\left({\text{k}}_{\text{i}}-1\right)}\right)$$

The clustering coefficient reflects the function of the local structure. As a result, it can be understood that when some error occurs in the network, the clustering coefficient reflects the repairability of the network. When a node has lost its function, the adjacent nodes can remain intact.

(4) $$\begin{array}{r} E_{loc\text{\_}i}=\frac{1}{n_{gi}\left(n_{gi}-1\right)}\underset{i,h\in gi}{\sum }\frac{1}{L_{j,h}} \end{array}$$

(5) $$\begin{array}{r} E_{loc}=\frac{1}{n}\underset{i\in G}{\sum }E_{loc\text{\_}i} \end{array}$$

(6) $$\begin{array}{r} E_{glob\text{\_}i}=\frac{1}{n-1}\underset{i\neq j\in G}{\sum }\frac{1}{L_{i,j}},E_{glob}=\frac{1}{n}\underset{i\in G}{\sum }E_{glob\text{\_}i} \end{array}$$

*E*_*loc*_*i*_ and *E*_*loc*_ are used to represent the local structure of node *i* and the network, where *gi* is the sub-network composed of node *i* and its connected nodes. The former reflects the degree of closeness of node *i* to the neighboring nodes, and the latter reflects the stability of the whole system, as shown in equation (4) and equation (5). Global efficiency, equation (6), is a good indicator to measure the transmission of information between parallel systems. Higher global efficiency is related to better transmission of information.

### Statistical Analysis

Statistical analysis was performed using the statistical software IBM SPSS 19.0, and the measurement data were analyzed by mean ± standard deviation (22, 23).

#### Comparison of Psychological Scale Scores Between Groups

Psychological cognitive outcomes of the mTBI group and the healthy control group were analyzed by multivariate covariance analysis after corrected for age, sex, and IQ to find out whether there is executive function change in mTBI group.

#### Correlation Analysis of Psychometric Scores and fMRI Processing

In order to explore whether the change in the brain network structure causes a change of psychological cognition, Pearson correlation analysis was performed on regions with abnormal functional parameters and various psychological cognition results after age, gender, and IQ correction. It is confirmed that the difference was statistically significant when *P* < 0.05. The results of Alff, ReHo, and graph theory were statistically analyzed with a two-sample *t*-test, this statistical method being the most appropriate and efficient way for experiments with only two groups of fMRI data (24).

## Results

### ReHo Analysis Result

**Figure 3:**
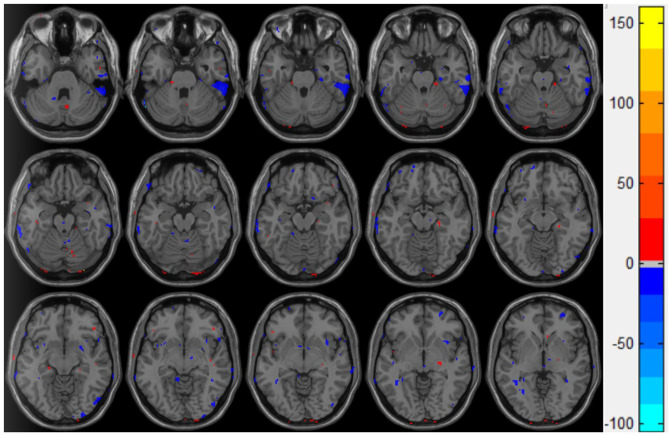
Double *t*-test of the ReHo value in the mTBI group and the control group: the blue part indicates that the ReHo value of the mTBI group is significantly lower than that of the normal group.

The ReHo value of the control group was significantly higher than that of the injury group in the following brain regions: the inferior temporal gyrus and the cerebellum superior, as shown in Figure 3.

### ALFF Analysis

**Figure 4:**
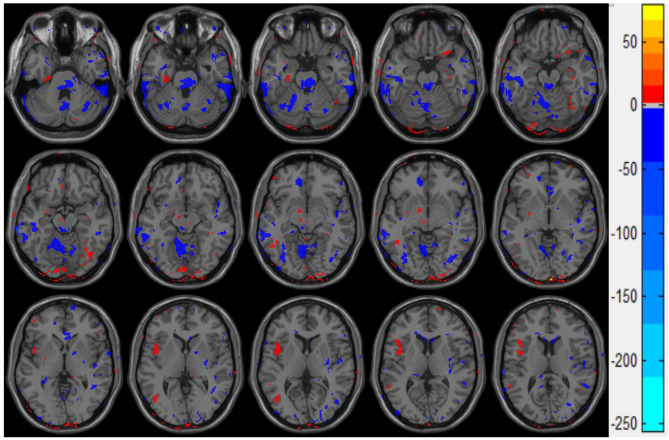
Double sample *t*-test results of ALFF of the mTBI group and control group. The red part indicates that the ALFF value of mTBI group is significantly higher than that of the control group.

Figure 4 presents the statistical two-sample *t*-test results of the ALFF between the mTBI group and the control group, the ALFF in the left insula, the cerebellum inferior, and the left middle occipital gyrus of the mTBI group being significantly higher than those in the normal group (Table 3). These results indicate that these three brain regions have a certain degree of dysfunction, which is usually observed in the brains of Parkinson's disease patients (25).

**Table 3:** The ALFF values in the mTBI group compared with those in the control group.

**Brain region**	**Peak ALFF**	**Location**		
		**X**	**Y**	**Z**
Insula_L	4.01	−44	6	6
Cerebellum_7b_R	2.64	16	−80	−49
Occipital_MID_L	3.55	−44	−68	6

### Clustering Coefficients, Local Efficiency, and Global Efficiency

**Figure 5:**
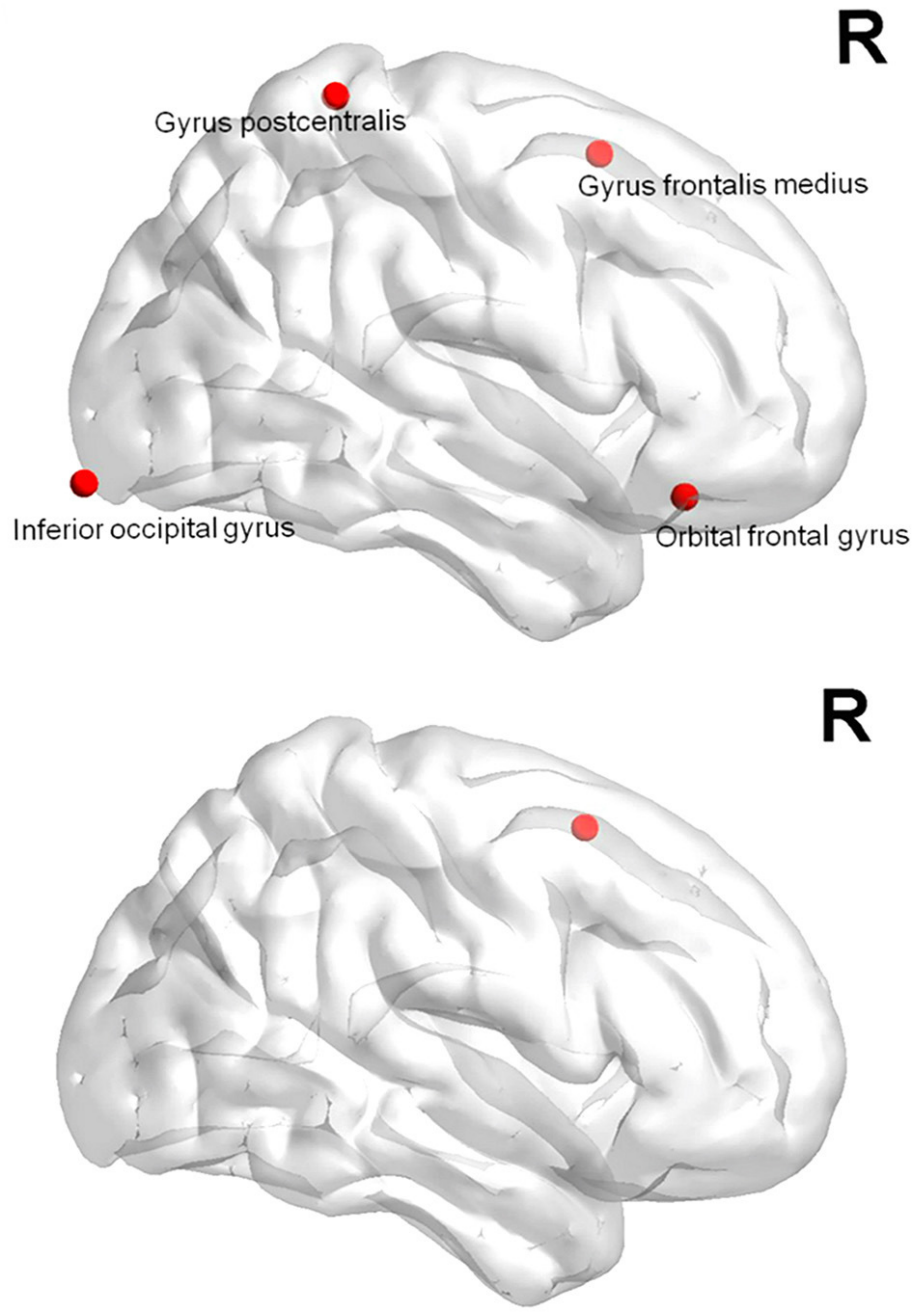
The decrease of clustering coefficients and local efficiency in the mTBI group.

In the mTBI group, the clustering coefficients of the right superior orbital frontal gyrus, the right middle frontal gyrus, the left inferior frontal triangle, and the right inferior occipital gyrus significantly decreased (Figure 5). And the clustering coefficients of the nodes in the right central posterior gyrus increased significantly (*p* < 0.05). Compared with the control group, the local efficiency of the left middle frontal gyrus of the mTBI group was significantly decreased (*p* < 0.05).

**Figure 6:**
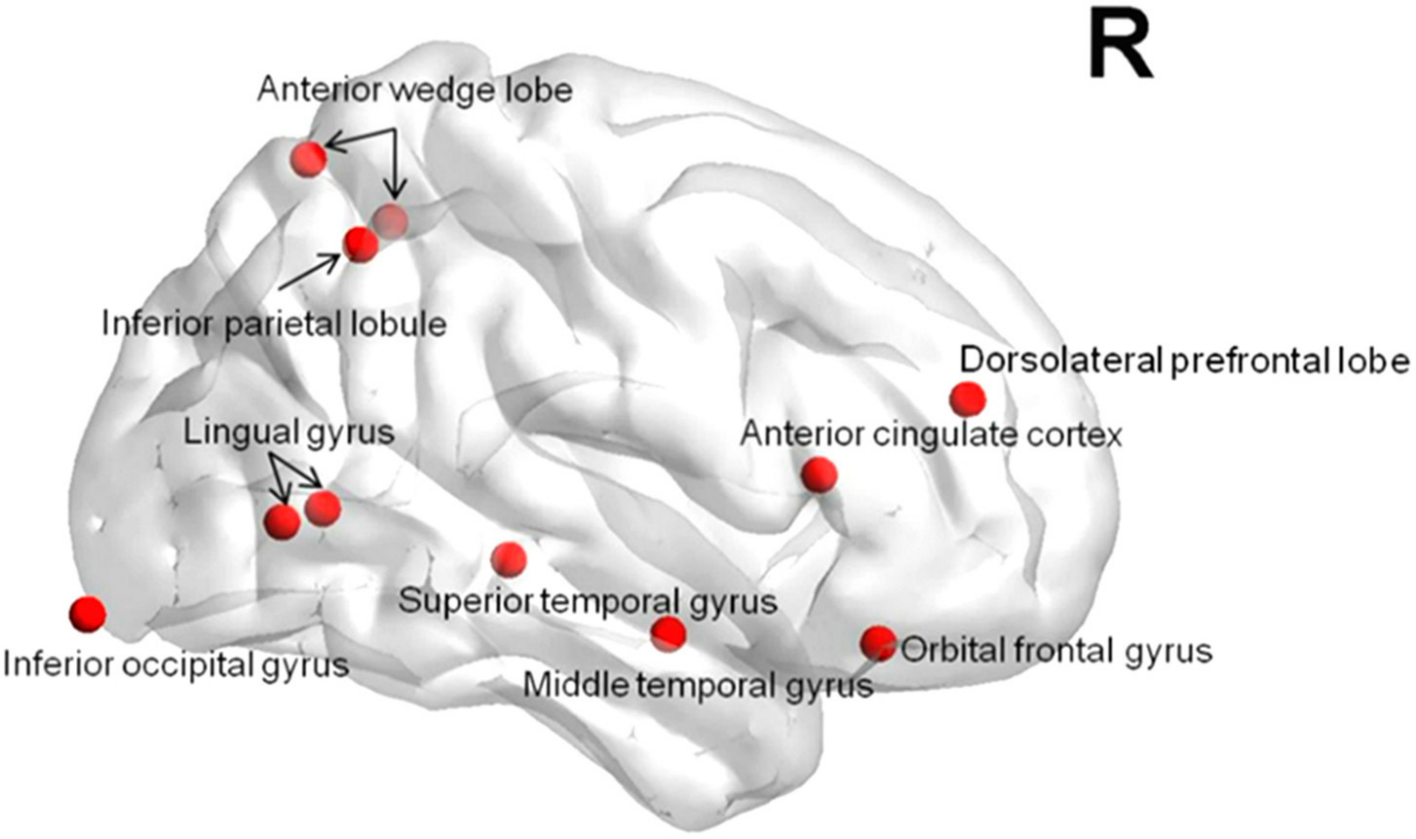
The decrease of nodes global efficiency in the mTBI group.

The global efficiency of nodes in the mTBI group significantly decreased in the right orbital frontal gyrus, the right anterior wedge lobe, the right superior temporal gyrus, the right middle temporal gyrus, the right dorsolateral pre-frontal cortex, the right anterior cingulate gyrus, the right suboccipital gyrus, the right gyrus lingualis, and the right inferior parietal lobe, as shown in Figure 6 (*p* < 0.05).

### Correlation Analysis of Psychometric Scores and fMRI Processing

**Figure 7:**
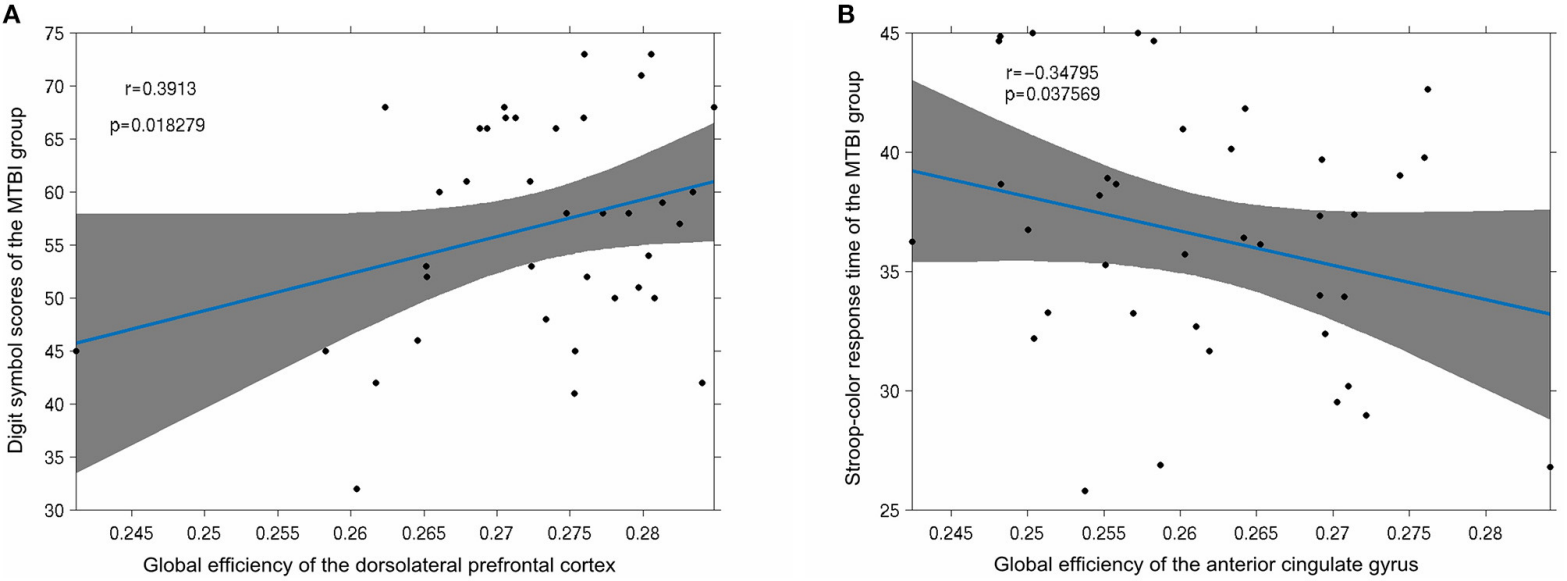
**(A)** Significant positive correlation was observed between the digit symbol scores of the mTBI group and the node global efficiency of the dorsolateral pre-frontal cortex (*p* < 0.05); **(B)** the stroop-color response time of the mTBI group was significantly negatively correlated with the global efficiency of the anterior cingulate gyrus (*p* < 0.05).

In this study, there was a strong positive correlation between global efficiency and the digit sign score (*p* < 0.05) of the dorsolateral pre-frontal cortex of the mTBI group (Figure 7A). The global efficiency of the anterior cingulate gyrus was strongly negatively correlated with the response time of the stroop-color test (*p* < 0.05; Figure 7B).

## Discussion

This study focused on investigating the altered local and global dysfunctional region to reveal the pathology of mTBI with multimode analysis, including ReHo, ALFF with graph theory, and neuropsychological outcomes. In our study, the main findings are as follows: both executive and kinematics dysfunction in mTBI patients as well as neuropsychological outcomes in acute phase existed, and significant differences in structure connections, ReHo, and ALFF in some brain regions between mTBI patients and control group could be found.

ReHo's statistical results showed that compared with the control group, the inferior temporal gyrus and the upper cerebellum of the brain injury group were significantly reduced. This result is consistent with the movement control, muscle tension control, sensory perception, micro-motor coordination, posture, and gait changes in patients with mTBI after injury (26, 27). It is also consistent with the difference in the kinematic test scores (Table 2) we observed in this study: the inferior temporal gyrus is mainly responsible for cognitive learning of objects. According to the statistical results of ALFF, the abnormal brain function in the brain injury group occurred in the left insula, the cerebellum inferior, and the left middle occipital gyrus. The middle occipital gyrus is the center of the visual cortex, and these abnormalities usually lead to visual disturbances, as well as memory and motor perception disturbances. This is in line with the clinical observation that mTBI patients tend to see double images in the acute phase and suffer from memory lapses. While the abnormal ALFF of the insula indicates the dysfunction of emotional adjustment and addiction, there's also a retrospective study of mTBI (28–32) that reported that patients who suffered mTBI in their youth were more likely to have emotional disorders and addiction problems.

Abnormal structural connections in the brain indicate the possibility of various types of dysfunction, and our study mainly presents the cerebellar-temporal lobe and basal ganglia-cerebellar circuits. Clinical studies have reported that mTBI patients usually suffer motor dysfunction and abnormal executive function in the acute phase, which is consistent with the results of this study in the cerebellar-temporal lobe structural connectivity abnormalities. Moreover, the basal ganglia are cooperatively regulated by the cerebral cortex and the cerebellum, and voluntary motor muscle tension and postural reflex also participate in the regulation of complex behaviors. The abnormalities of basilar-cerebellar structural connections obtained in this study need to be further explored in relation to clinical symptoms. And the limitations of this study include the lack of long-term follow-up data and convalescence stage fMRI analysis, which deserves to be further investigated.

The behavioral results of this study found that the mTBI patients performed worse on the number symbol test and the Stroop test than the control group, which reflects the discrepancy in attention and short-term test of memory, perceptual discrimination, writing speed, and the efficiency of association and information processing. Our experimental results show that patients with mTBI cannot process external information normally and give appropriate feedback during the acute phase: it took a while to recover their working memory and work efficiency. At the same time, mTBI patients take longer on the Stroop test compared with the control group. The Stroop mainly evaluates attention control and attention execution ability of the subjects, suggesting that mTBI patients have control and executive dysfunction in the acute phase. These cognitive abnormalities are related to our analysis in graph theory, as shown in Figure 7.

## Conclusions

Compared with the control group, the acute phase mTBI group performed poorly in the DSTT and Stroop test, indicating a dysfunction among mTBI patients in movement speed, association, information processing speed, attention, and short-term memory. Meanwhile, abnormal multimode rsMRI parameters in some brain regions of acute mTBI patients are correlated with executive function performance, indicating that changes in local brain regions may be the cause of the decline in information processing speed and executive function. In conclusion, multimode analysis of mTBI patients based on rs-fMRI, including ALFF, ReHo, graph theory, and neuropsychological outcomes, can be used for more objective and accurate brain region assessment of acute mTBI.

## Data Availability Statement

The raw data supporting the conclusions of this article will be made available by the authors, without undue reservation.

## Ethics Statement

The study was approved by the IRB of Third Xiangya Hospital, Central South University, and the study on humans was carried out in accordance with the relevant Measures for the Ethical Review of Biomedical Research Involving Humans (China, 2016). All participants in the study signed an informed consent form.

## Author Contributions

JS and NL contributed to the conceptualization of the study, supervision, and formal analysis. JT and XD contributed to the conceptualization, data curation, write-up, and editing of the article. NL contributed to the supervision, conceptualization, data curation, and write-up. All authors have read and agreed to the published version of the manuscript.

## Conflict of Interest

The authors declare that the research was conducted in the absence of any commercial or financial relationships that could be construed as a potential conflict of interest.
